# Lipopolysaccharide-Induced Weakness in the Preterm Diaphragm Is Associated with Mitochondrial Electron Transport Chain Dysfunction and Oxidative Stress

**DOI:** 10.1371/journal.pone.0073457

**Published:** 2013-09-06

**Authors:** Yong Song, Gavin J. Pinniger, Anthony J. Bakker, Timothy J. M. Moss, Peter B. Noble, Clare A. Berry, Jane J. Pillow

**Affiliations:** 1 School of Anatomy, Physiology and Human Biology, The University of Western Australia, Perth, Western Australia, Australia; 2 Centre for Neonatal Research and Education, School of Paediatrics and Child Health, The University of Western Australia, Perth, Western Australia, Australia; 3 The Ritchie Centre, Monash Institute of Medical Research, and Department of Obstetrics & Gynaecology, Monash University, Melbourne, Victoria, Australia; 4 Women and Newborns Health Service, c/−King Edward Memorial and Princess Margaret Hospitals, Perth, Western Australia, Australia; University of Kentucky, United States of America

## Abstract

Diaphragmatic contractility is reduced in preterm lambs after lipopolysaccharide (LPS) exposure *in utero*. The mechanism of impaired fetal diaphragm contractility after LPS exposure is unknown. We hypothesise that *in utero* exposure to LPS induces a deficiency of mitochondrial complex activity and oxidative damage in the fetal diaphragm. To test this hypothesis, we used a well-established preterm ovine model of chorioamnionitis: Pregnant ewes received intra-amniotic (IA) saline or 10 mg LPS, at 2 d or 7 d prior to surgical delivery at 121 d GA (term = 150 d). The fetus was killed humanely immediately after delivery for tissue sampling. Mitochondrial fractions were prepared from the isolated diaphragm and mitochondrial electron transfer chain activities were evaluated using enzymatic assays. Oxidative stress was investigated by quantifying mitochondrial oxidative protein levels and determining antioxidant gene and protein (catalase, superoxide dismutase 2 and glutathione peroxidase 1) expression. The activity of the erythroid 2-related factor 2 (Nrf2)-mediated antioxidant signalling pathway was examined by quantifying the Nrf2 protein content of cell lysate and nuclear extract. A 2 d LPS exposure *in utero* significantly decreased electron transfer chain complex II and IV activity (p<0.05). A 7 d LPS exposure inhibited superoxide dismutase 2 and catalase expression at gene and protein levels, and Nrf2 pathway activity (p<0.05) compared with control and 2 d LPS groups, respectively. Diaphragm mitochondria accumulated oxidised protein after a 7 d LPS exposure. We conclude that intrauterine exposure to LPS induces mitochondrial oxidative stress and electron chain dysfunction in the fetal diaphragm, that is further exacerbated by impairment of the antioxidant signalling pathway and decreased antioxidant activity.

## Introduction

Compromised initiation and maintenance of respiration in the premature infant is usually attributed to immature lung development. However, neonatal respiratory failure may be due in part to insufficient development of the principal respiratory muscle (diaphragm) *in utero*. Chorioamnionitis, inflammation of the placental and fetal membranes, is associated with preterm birth and can induce a fetal inflammatory response [1]. Recently, we showed that a 2 d or 7 d exposure to intrauterine lipopolysaccharide (LPS) reduced diaphragm contractile force by 30% in preterm lambs [2]. Therefore, exposure to an intrauterine inflammatory insult may compromise the integrity of the diaphragm at delivery and critically influence the susceptibility of the newborn to development of respiratory failure after birth.

Studies of muscle weakness in adult models of muscle atrophy show that muscle fibre injury is associated with oxidative stress and/or that mitochondrial dysfunction and increased mitochondrial production of reactive oxygen species (ROS) play a key role in triggering the atrophic signals [3]–[5]. The concept of oxidative stress-induced muscle weakness is supported by evidence that mitochondria-targeted antioxidants attenuated immobilization-induced increases in mitochondrial ROS production and prevented oxidative stress, protease activation, and myofiber atrophy [6]. Additionally, ROS have direct deleterious effects on muscle contractile function by altering myofibrillar Ca^2+^ sensitivity and cross-bridge kinetics [7], independent of accelerated proteolysis.

The intermyofibrillar space of the preterm diaphragm has many large mitochondria compared with the adult diaphragm, suggesting that the preterm diaphragm is highly oxidative despite the predominance of Type II fibres during this stage of development [8]. Despite the mitochondrial predominance, the antioxidant defence system is not fully developed in the diaphragm until full term [9]. Moreover, during delivery, diaphragmatic ROS accumulate dramatically and potentially overwhelm the compromised preterm oxidant defensive system [10]. The imbalance between oxidative capacity and antioxidant defence means that the newborn preterm infant is especially prone to oxidative stress.

Chorioamnionitis promotes oxidative stress. We showed previously that a 7 d exposure to intra-amniotic (IA) LPS increases circulating neutrophils [11], and causes systemic oxidative stress in fetal lambs [12]. The mitochondrial electron transport chain is the major source of intracellular ROS and is also susceptible to damage initiated by ROS [13], [14]. Thus, systemic oxidative stress may promote excessive ROS production that in turn impairs mitochondrial electron transport chain function and induces further ROS emission from mitochondria, initiating a vicious cycle.

In this study, we hypothesised that preterm diaphragm dysfunction after fetal exposure to an *in utero* inflammatory stimulus would be associated with down-regulation of mitochondrial electron transport chain activity and oxidative damage, and that the associated redox disturbances were implicated in the signalling events of preterm diaphragm dysfunction. We aimed to identify the alterations in mitochondrial enzyme activity, protein oxidation, antioxidant activity and signalling in the preterm fetal diaphragm following exposure to IA LPS.

## Materials and Methods

### Animals and Experimental Design

This study was approved by the Animal Ethics Committee of The University of Western Australia (Permit Number: RA/3/100/1000). Time-mated ewes with singleton fetuses were randomly assigned to one of two groups. One group received an IA injection of LPS (10 mg *Escherichia coli* 055:B5, Sigma Chemical, St. Louis, MO) at either 7 d or 2 d prior to surgical delivery at 121 d gestational age (GA), respectively (term = ∼ 150 d). Controls received an equivalent IA injection of saline either 7 d or 2 d prior to delivery. The fetus was humanely killed with pentobarbitone (150 mg/kg IV, Pitman-Moore, Australia) at delivery. Immediately after delivery, the costal right hemidiaphragm was dissected, frozen in liquid nitrogen and stored at –80°C.

### Preparation of Isolated Mitochondria and Diaphragm Homogenates

Mitochondrial isolations were performed at 4°C using a Mitochondria Isolation Kit for Tissue (Pierce, Rockford, USA) according to manufacturer’s instructions. Briefly, 100 mg of homogenized diaphragm tissue was centrifuged at 1 000 g for 3 minutes at 4°C and the resulting pellet was suspended in 800 µl BSA/Reagent A Solution. After 2 min incubation, 10 µl Mitochondria Isolation Reagent B and 800 µl Mitochondria Isolation Reagent C were added. Following centrifugation at 700 g for 10 min at 4°C, the supernatant was subjected to further centrifugation at 3 000 g for 15 min at 4°C. Subsequently, 500 µl washing buffer was added to the mitochondrial pellet, and the mixture was surface washed by centrifuging at 12 000 g for 5 min. The mitochondrial pellet was maintained at 4°C before subsequent processing.

The whole-cell lysate was prepared as described previously [9]. Nuclear protein fractions were prepared from costal segments of the diaphragm using NE-PER® Nuclear and Cytoplasmic Extraction Reagents with inclusion of Halt Protease Inhibitor Cocktail (Thermo Scientific, Massachusetts, USA), according to manufacturer’s instructions. Both extracts were assayed using the Bradford method (Sigma, Sydney, Australia). 50 µg of protein from each fraction were separated by Western blot for erythroid 2-related factor 2 (Nrf2) signalling (described below).

### Enzymatic Assays for Mitochondrial Respiratory Complex Activity

Mitochondrial respiratory chain complex activity was analysed in accordance with the protocol described by Kavazis *et al.* [15]. Briefly, complex I (NADH dehydrogenase, EC 1.6.5.3) enzyme activity was measured as a decline in absorbance from NADH oxidation by decylubiquinone before and after adding rotenone. Complex II (succinate dehydrogenase, EC 1.3.5.1) activity was determined as a function of the decrease in absorbance from 2, 6-dichloroindophenol reduction. Complex III (ubiquinol cytochrome c oxidoreductase, EC 1.10.2.2) activity was calculated as a function of the increase in absorbance from cytochrome c reduction. Complex IV (cytochrome c oxidoreductase, EC 1.9.3.1) activity was measured as a function of the decrease in absorbance from cytochrome c oxidation. Mitochondrial complex activity was normalized to whole mitochondrial protein content and expressed as arbitrary units.

### Measurement of Reactive Carbonyl Derivatives

Oxidized proteins in diaphragm mitochondria were quantified using OxyBlot™ Protein Oxidation Detection Kit (Millipore**,** Billerica, USA). The carbonyl groups in the protein side chains of proteins were derivatized to 2, 4-dinitrophyenylhydrazone (DNP) and subsequently separated using sodium dodecyl sulfate–polyacrylamide gel electrophoresis (SDS-PAGE). After electrophoresis, the proteins were transferred to nitrocellulose membranes. The resulting membranes were probed with specific antibodies to the DNP for 1 h, followed by thoroughly washing and adding secondary antibody coupled to horseradish peroxidase. The membranes were then treated with chemiluminescent reagents and the oxidative status of samples was analysed quantitatively by comparison of the signal intensity of the total protein.

### Gene Expression Assay

Total RNA was isolated from diaphragm tissue samples and subsequently used to synthesize cDNA. Expression of catalase, superoxide dismutase 2 (SOD2) and glutathione peroxidase 1 (GPX1) was measured using Rotor-Gene SYBR Green PCR Kit (Qiagen Pty, Doncaster, Australia). The primers for catalase were designed on the ovine gene sequence (GQ421282) to target the specific mRNA regions (Forward: CGCCTGTGTGAGAACATTGC; Reverse: TGCTGCACATAGGTGTGAAC), whilst Nrf2 primers (Forward: CAAAATGACAAGCTGGCTGA; Reverse: AAATGTGGGCTGCAGTTACC) were designed to target bovine-specific mRNA range of *Nrf2* gene (AB162435) due to high homology between ovine and bovine genomic sequences. The other primers used in this study were described previously [9]. For normalization, the values for the target sequence were calculated in relation to the arithmetic median of the values obtained from average of two housekeeping genes (18 S RNA and β-actin) using 2^−ΔΔCT^ method and are presented relative to values in the control group.

### Western Blot

Mitochondrial or cellular/nuclear extracts were separated by 12% SDS-PAGE and transferred to nitrocellulose membranes (30 V overnight at 4°C). The resulting membranes for mitochondrial proteins were further stained with Ponceau S and analysed to verify equal loading and transfer. After blocking in PBS containing 5% skimmed milk, the membranes were incubated with primary antibodies against catalase (1∶5000), SOD2 (1∶5000), GPX1 (1∶4000), voltage-dependent anion channel 1 (VDAC1, 1∶1000), Nrf2 (1∶1000), and α-Tubulin (1∶2000) for 2 h at room temperature. The antibodies used were all purchased from Abcam (Cambridge, MA, USA) except the anti α-Tubulin antibody, which was purchased from Cell Signalling Technology (Carlsbad CA USA). Bound antibodies were detected with anti-rabbit immunoglobulin conjugated with horseradish peroxidise (1∶2000 dilution) for 1 h. After adding a chemiluminescent substrate (Thermo Scientific, Massachusetts, USA), immunoreactive protein signals were detected and quantified by computerized image analysis (ImageQuant™ 350, GE Healthcare, Little Chalfont, UK). The values for mitochondrial and cellular/nuclear proteins were normalized into VDAC1 and α-Tubulin abundance respectively.

### Statistical Analysis

Sigmaplot (version 11.0, Systat Software Inc, San Jose, USA) was used for statistical analysis. Differences between groups (Control, 2 d LPS, 7 d LPS) were assessed using one-way ANOVA with a Tukey honestly significant difference (HSD) test implemented as post hoc analysis. Nonparametric data were analysed using ANOVA on ranks. Pearson correlation analysis was used to determine any association between Nrf2 activity and antioxidant gene expression. P<0.05 was considered statistically significant. Data are presented as mean (SEM) or median (range).

## Results

### Mitochondrial Electron Transport Chain Complex Activities

**Figure 1:**
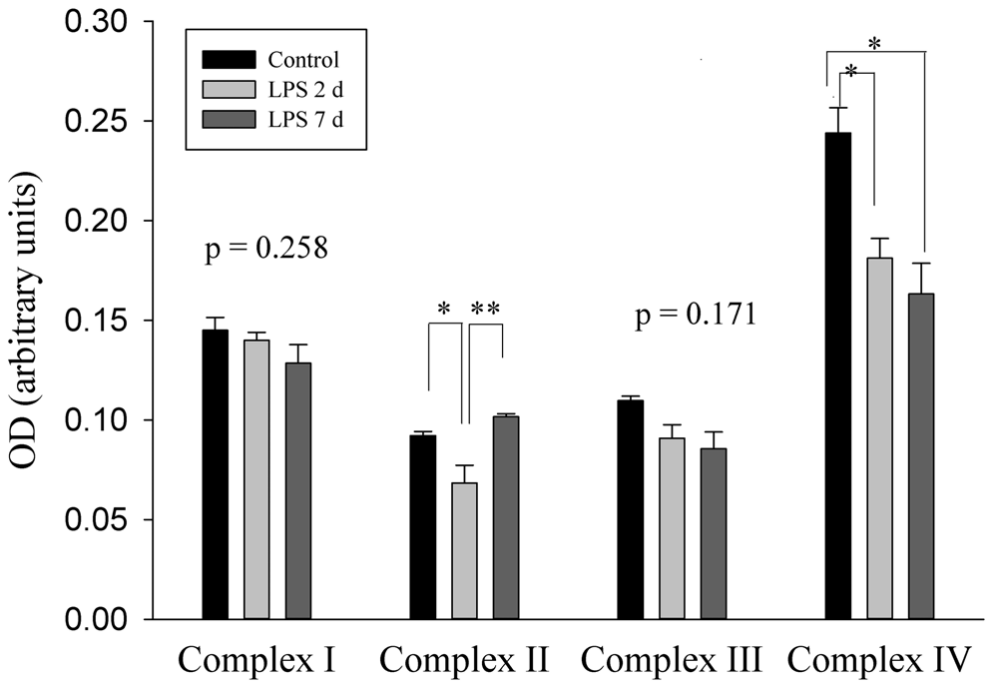
Mitochondrial Respiratory Chain Complex Activity of Fetal Diaphragm. Graph shows activities of mitochondrial complex I-IV in fetal diaphragm for saline (n = 3) and 2 d (n = 7) and 7 d (n = 3) LPS treated groups. Values are Mean (SEM). *indicates p<0.05, while **indicates p≤0.01.

The activities of diaphragmatic mitochondria complexes I, II, III, and IV were evaluated by spectrophotometric methods (Figure 1). Complex I and III activities showed no significant change after LPS treatment, although tended to decrease with increasing duration of LPS exposure. Complex II activity decreased after 2 d LPS exposure (p<0.05), with evident partial recovery of activity after a 7 d LPS exposure (p = 0.01). In contrast, the activity of complex IV was diminished by 26% after 2 d LPS with further loss of activity (33% decrease) in the 7 d LPS group compared with the controls (p<0.05).

### Protein Oxidation in Diaphragmatic Mitochondria

**Figure 2:**
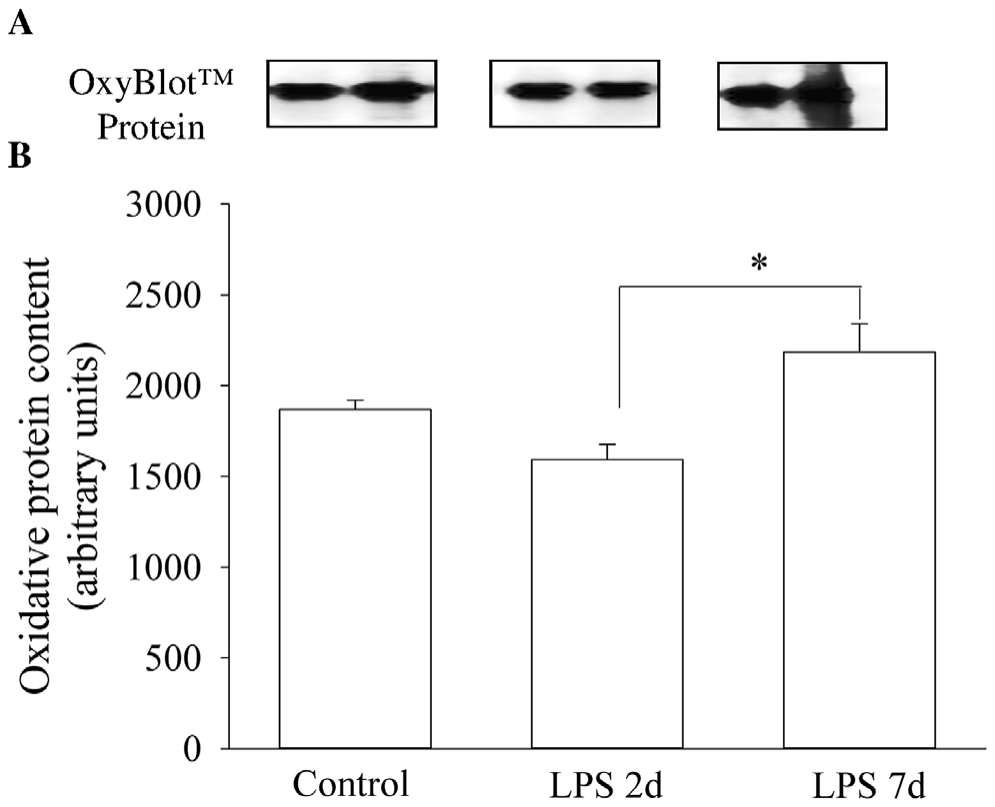
Quantification of Oxidized Protein in Diaphragm Mitochondria. Western blots illustrate protein oxidation using representative samples from each group (A). Graph shows the reactive carbonyl derivatives content in the isolated diaphragmatic mitochondria in control animals (n = 3) and animals exposed to LPS for 2 d (n = 7) or 7 d (n = 3) (B). Values are Mean (SEM). *indicates p<0.05.

Changes in reactive carbonyl derivatives were measured as a hallmark of the oxidation status of protein in diaphragmatic mitochondria of the experimental groups. Compared with the control group, 2 d and 7 d LPS did not result in a significant change in the reactive carbonyl derivatives. However, relative to 2 d LPS exposure, there was a significant increase in reactive carbonyl derivatives in diaphragmatic mitochondria after 7 d LPS treatment (Figure 2).

### Antioxidant Gene Expression

**Figure 3:**
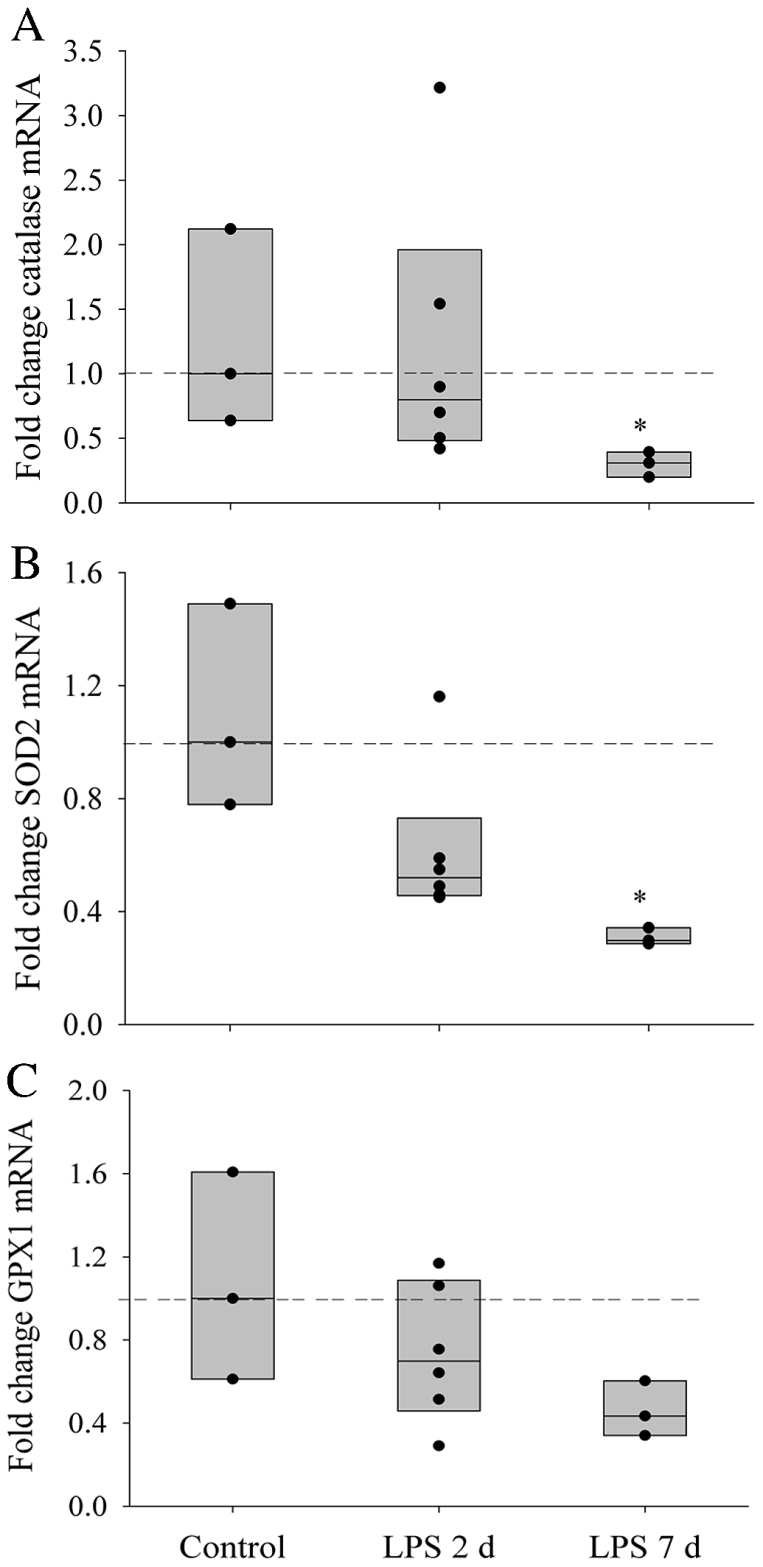
Expression of Antioxidant Genes. Graphs show catalase (A), SOD2 (B) and GPX1 (C), mRNA expression in preterm diaphragm mitochondria after either saline (n = 3), 2 d (n = 6) or 7 d (n = 3) LPS treatment *in utero*. Values are Median. Horizontal dashed bar indicates median of reference (control) group. *indicates p<0.05 compared with control group.

Both catalase and SOD2 gene expression decreased as LPS exposure time increased, with a 4-fold reduction in the 7 d LPS group compared to control (p<0.05) (Figure 3A and 3B). The GPX1 mRNA level also exhibited a decreasing trend with increasing duration of fetal exposure to IA LPS (p = 0.151), although there was no significant difference observed in any two groups (Figure 3C).

### Mitochondrial Antioxidant Enzymes

**Figure 4:**
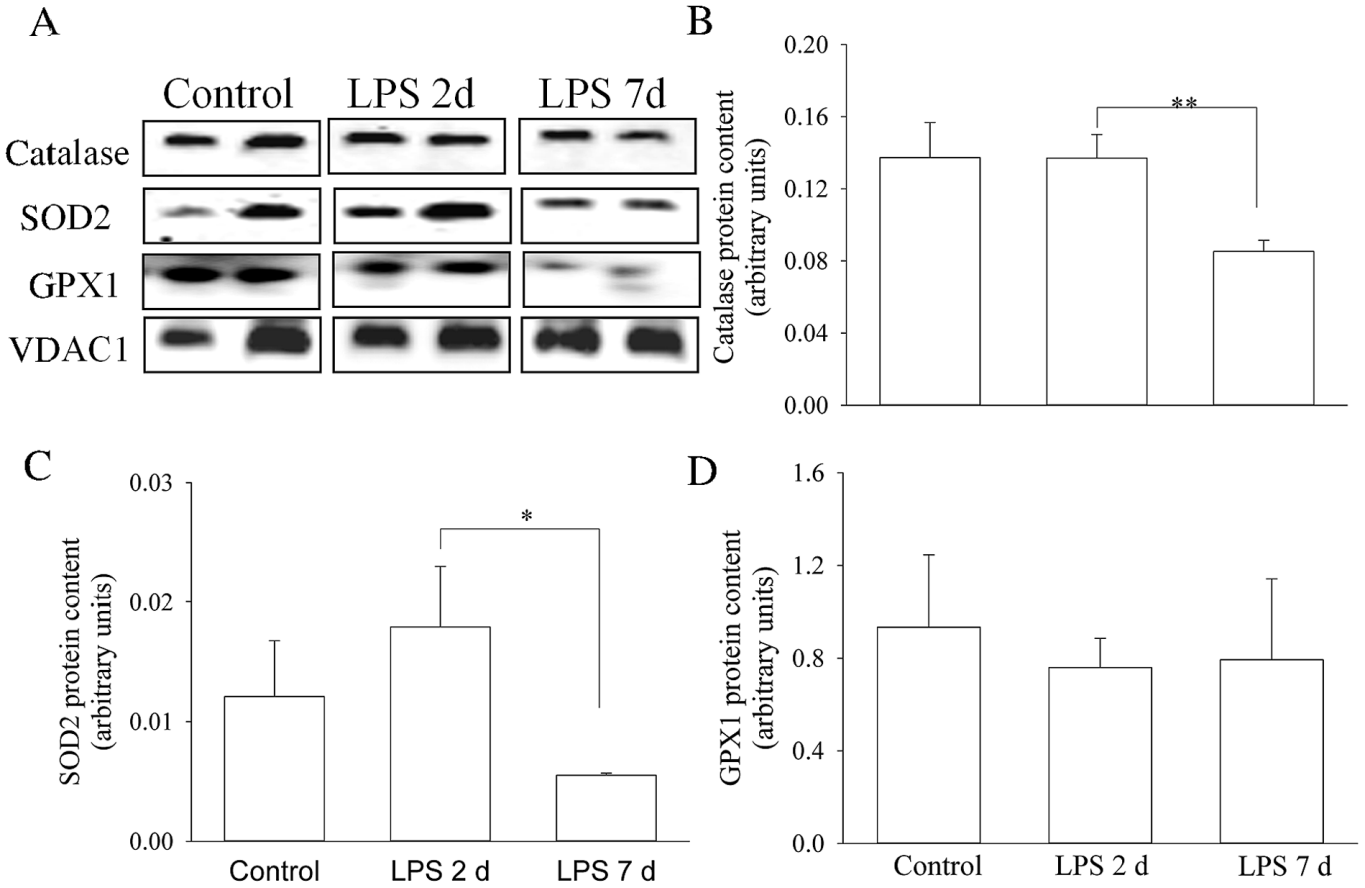
Antioxidant Protein Content in Diaphragm Mitochondria. Western blots illustrate expression of antioxidant proteins using representative samples from the control group (n = 3), and the 2 d (n = 7) and 7 d (n = 3) LPS groups (A). Graphs show protein contents of catalase (B), SOD2 (C) and GPX1 (D) in saline and LPS treated groups. Values are Mean (SEM). *indicates p<0.05.

A 2 d or 7 d *in utero* LPS exposure did not cause a distinct change in antioxidant enzyme content in diaphragmatic mitochondria compared with fetal controls. Nevertheless, compared with 2 d exposure, catalase and SOD2 proteins decreased significantly after a 7 d *in utero* exposure to LPS (p<0.01 and p<0.05, respectively) (Figure 4A–C). Mitochondrial GPX1 level was unchanged by LPS exposure at either 2 d or 7 d prior to delivery at 121 d compared with fetal controls (p>0.05) (Figure 4D).

### Nrf2 Signalling

**Figure 5:**
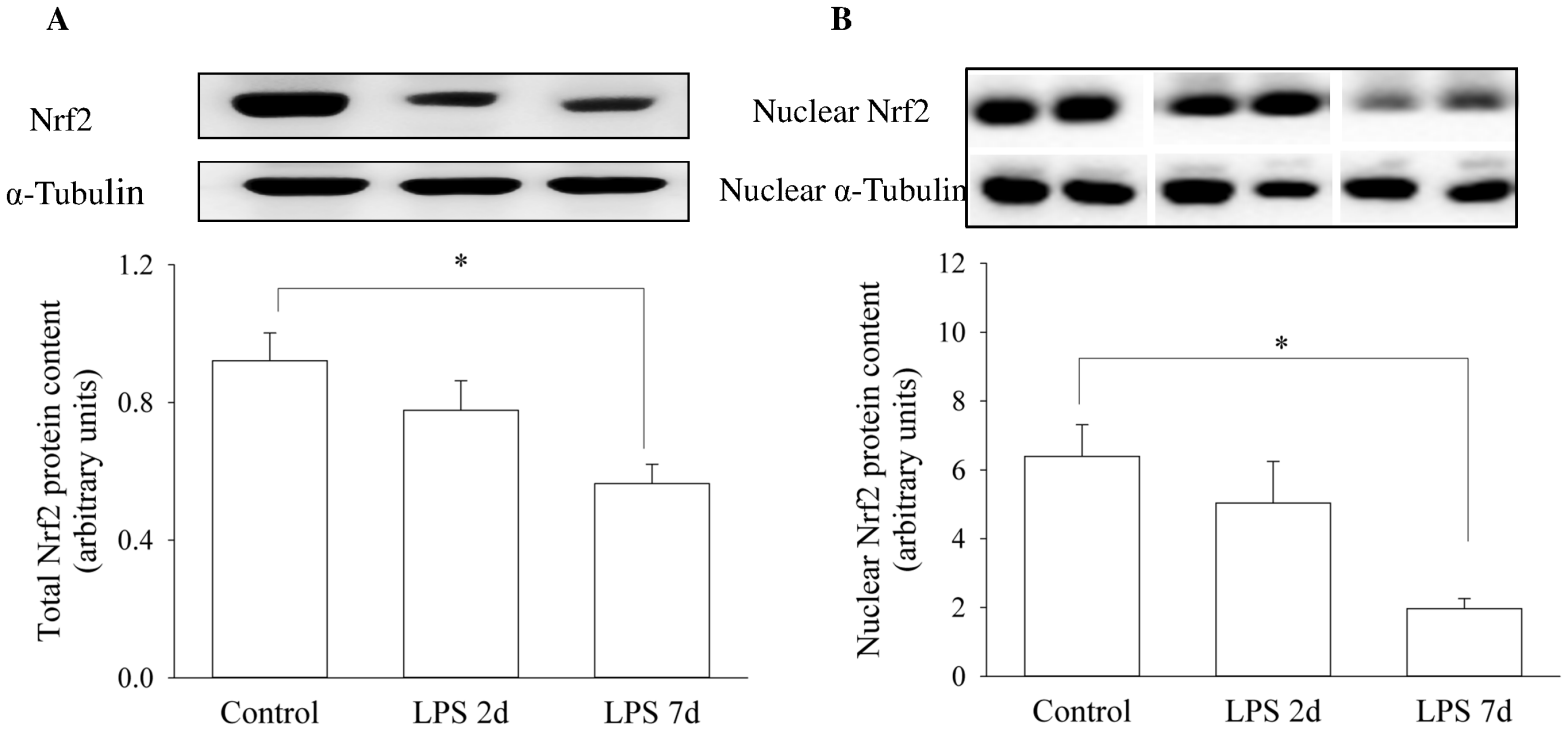
Activity of Nrf2 Signalling Molecule after LPS Exposure. Western blots illustrate Nrf2 protein concentration in whole cell lysate and nuclear fraction using representative samples from each group above the graphs. Graph A shows Nrf2 protein content in cell lysate in the control (n = 5) and 2 d (n = 7) and 7 d (n = 7) LPS exposure groups. Graph B shows nuclear Nrf2 protein content for saline (n = 5), 2 d (n = 5) and 7 d (n = 5) LPS challenged groups. Values are Mean (SEM). *indicates p<0.05.

**Figure 6:**
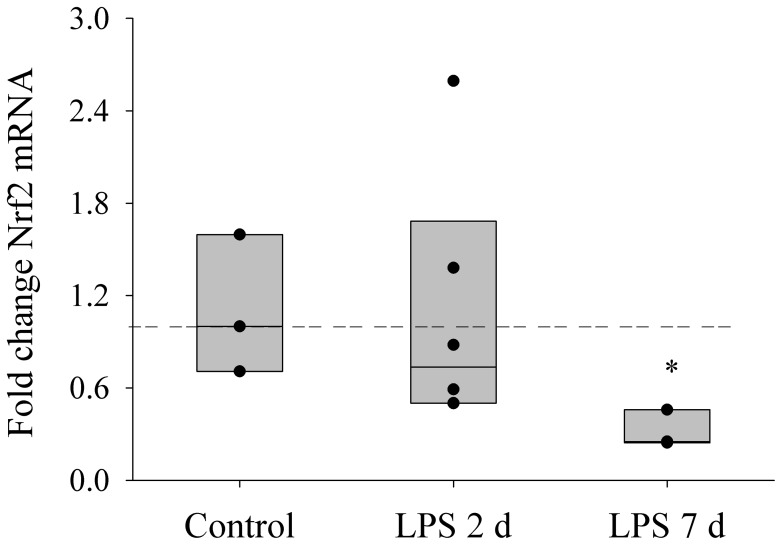
*Nrf2* Gene Expression. Graphs show Nrf2 mRNA expression in preterm diaphragm mitochondria after either saline (n = 3), 2 d (n = 6) or 7 d (n = 3) LPS treatment *in utero*. Values are Median. Horizontal dashed bar indicates median of reference (control) group. *indicates p<0.05 compared with control group.

To further investigate whether the Nrf2 signalling pathway is responsible for the down-regulation of the antioxidant genes, we examined Nrf2 protein content in cell lysate and nuclear fractions in the different experimental groups. Nrf2 protein experienced a gradual fall with increasing *in utero* LPS exposure time, and there was a significant reduction in both total (Figure 5A) and nuclear (Figure 5B) Nrf2 protein levels in the 7 d LPS group (p<0.05). In accordance with the change of Nrf2 protein, *Nrf2* gene expression was significantly reduced after a 7 d *in utero* exposure to LPS (p<0.05) (Figure 6). In addition, protein content of cell lysate Nrf2 was significantly correlated with mRNA level of SOD2 (r = 0.845, p = 0.001) and GPX1 (r = 0.611, p<0.05), but not with catalase gene expression level.

## Discussion

We used an ovine fetal model to investigate whether mitochondrial electron transport chain dysfunction and oxidative stress are present in preterm diaphragm after acute exposure to intrauterine inflammation. Our data show that IA LPS injection induced diaphragm mitochondrial oxidative stress and electron chain dysfunction after a 2 d or 7 d exposure in preterm lambs. Fetal capacity to overcome oxidative stress after a 7 d LPS treatment was impaired by down-regulation of the antioxidant gene and enzyme expression and accompanying Nrf2 signalling deficiency.

Mitochondrial dysfunction is evident in animal models of sepsis [16]–[19] and contributes to respiratory muscle weakness [5] and organ damage [20], [21]. The pathogenesis of sepsis-induced mitochondrial injury is associated with free radical generation and the release of a variety of exacerbating inflammatory mediators (e.g. TNF-α, IL-1β and IL-6) which directly or indirectly influence mitochondrial function and energy production [20]. Callahan et al reported that sepsis evoked down-regulation of diaphragm electron transport chain components [19], [22], which was attributed to sepsis-induced generation of free radicals [23]. Several research groups have reported that free radicals may directly affect electron transport chain activity [24] or induce mitochondrial protein depletion resulting in altered electron transport chain function during endotoxin-induced sepsis [23]. Increased ROS emission through electron leak from the respiratory chain in mitochondria may exacerbate mitochondrial impairment and induce mitochondrial oxidative stress. Since IA LPS injection promotes systemic oxidative stress and an inflammatory response in the preterm fetal lamb [12], [25], [26], we hypothesised that LPS-induced weakness in the preterm diaphragm would be associated with inhibition of mitochondrial complex activity and oxidative stress. This hypothesis is supported by our findings that a 2 d IA LPS exposure depresses the activity of electron transport chain complexes II and IV, and that a longer LPS exposure (7 d) increased oxidative modified protein content, compared with the 2 d exposure group.

An alternative possibility is that impaired mitochondrial complex activity enhanced the production of ROS and was primarily responsible for the oxidative stress that occurred after a 7 d *in utero* LPS exposure. The mitochondrial electron transport chain generates superoxide at complexes I, II, and III [14], [27]. Complex III is the principal site of ROS production by generating superoxide at the Qo site, resulting in superoxide accumulation in the intermembrane space or the matrix [27]–[29]. Although activity of complex III did not change, the pattern of decreasing complex III activity with increasing duration of fetal exposure to LPS was consistent with that observed for complex IV. Complex IV activity was significantly decreased in both 2 d and 7 d LPS groups. Complex IV (cytochrome *c*) has not been reported to generate ROS; however, cytochrome *c* participates in the generation of hydrogen peroxide by providing electrons to p66 Shc [30].

Oxidative stress occurs when the production of ROS overwhelms the scavenging capacity of antioxidant system. Under physiological conditions, an increase in ROS stimulates further antioxidant enzyme expression, increasing the capacity of the antioxidant defence system to maintain redox homeokinesis and protect cells from ROS-induced oxidative damage [31]. This adaptive response to accumulation of free radicals is also observed in diaphragm preparations weakened after exposure to controlled mechanical ventilation of diaphragm or soleus muscle preparations after immobilization [15], [32] and represents a cell-protective mechanism for managing oxidative stress.

Unlike the adult, the antioxidant system of the fetus is underdeveloped and susceptible to environmental insults resulting in oxidative stress during fetal development. Consequently, an increase in production of ROS may overwhelm the compromised preterm oxidant defensive system, promoting the development of oxidative stress. We examined gene expression and protein abundance of three primary mitochondrial antioxidant enzymes (catalase, SOD2 and GPX1) in the preterm lamb diaphragm to understand how antenatal exposure to an inflammatory stimulus impacted on redox status in the fetal diaphragm. Catalase, SOD2 and GPX1 usually act in a coordinated way against ROS-mediated damage. SOD can dismutate superoxide anions to hydrogen peroxide, which are subsequently converted to water and oxygen by catalase. Similarly, GPX1 utilizes reduced glutathione as a reducing equivalent to reduce hydrogen peroxide to form oxidized glutathione and water. We showed that a 7 d *in utero* LPS exposure inhibited catalase and SOD2 gene and protein expression, but not GPX1, reflecting a decrease in ROS scavenging which coincides with mitochondrial oxidative protein accumulation. Thus, down-regulation of antioxidant enzymes may be a secondary mechanism leading to mitochondrial oxidative stress in the preterm lamb.

Nrf2 transcriptional factor regulates the expression of antioxidant genes (e.g. catalase, SOD and GPX) through antioxidant response cis-elements [33], [34]. We analysed Nrf2 protein levels in the cell lysate and nuclear fraction of diaphragm muscle to investigate whether Nrf2 signalling participates in down-regulation of antioxidant genes after fetal exposure to IA LPS. Our finding that the nuclear expression of Nrf2 decreased with increasing duration of LPS exposure and the significant association between Nrf2 protein level and antioxidant gene expression is consistent with a central role of Nrf2 in down-regulation of antioxidant genes in the LPS-exposed fetus. Under non-stressed conditions, Nrf2 is sequestered in the cytoplasm as an inactive complex and constitutively degraded through the ubiquitin–proteasome system (UPS) by binding to Kelch-like ECH-associated protein 1 (Keap1). Oxidative or covalent modification of thiols in some cysteine residues of Keap1 lead to dissociation of Nrf2 from Keap1 and subsequently nuclear accumulation of Nrf2. However, the fall in nuclear Nrf2 with increasing duration of LPS exposure was likely due to Nrf2 deficiency rather than increased binding of Keap1 as there was a corresponding fall in the Nrf2 level in the whole cell lysate, with a significant reduction after a 7 d *in utero* LPS exposure. Nrf2 deficiency is thus likely to be responsible for down-regulation of antioxidant gene expression after 7 d LPS exposure. In a recent study, we showed that activation of UPS was observed after IA LPS exposure for 2 d [2], which does not fully account for Nrf2 deficiency. Given that an inflammatory stressor could influence fetal programming during gestational development, we further examined Nrf2 mRNA levels. Indeed Nrf2 gene expression was inhibited in the 7 d LPS group, indicating that *in utero* LPS exposure alters Nrf2 signalling via impairment at a transcriptional level.

Mitochondrial oxidative stress occurred after 7 d LPS exposure but not 2 d LPS exposure, which may represent a progressive change in mitochondrial function and redox signalling behaviour associated with increasing duration of LPS exposure. Arguably, the more premature infants may also have an increased susceptibility to an *in utero* inflammatory stimulus. Thus, different gestational age (and diaphragm developmental stage) at the time of LPS exposure may also be a critical factor in contributing to such difference. Our study is also limited by a low sample size with only three animals in each of the control and 7 d exposure groups for some studies. Larger studies are required to confirm and extend our present findings.

### Summary and Clinical Relevance

Inflammation of the placental and fetal membranes, known as chorioamnionitis, is strongly associated with preterm delivery. Exposure to an inflammatory stimulus *in utero* promotes fetal lung maturation, such that it reduces the severity of respiratory distress syndrome immediately after preterm birth. However, despite minimal severity of initial lung disease, many preterm infants subsequently exhibit respiratory insufficiency, that may be due in part to weakness of the respiratory muscles exacerbated by a fetal inflammatory response. Overall, this study shows that an *in utero* LPS exposure is associated with mitochondrial electron transport chain dysfunction and oxidative stress in the preterm fetal diaphragm, and dysregulation of the Nrf2-mediated antioxidant response. Impaired mitochondrial enzyme activity and oxidative stress may explain in part, our earlier finding of impaired contractile function after *in utero* LPS exposure [2].

Our observation of dysregulation of the Nrf2-mediated antioxidant response has implications for future therapeutic interventions. Cellular antioxidant defences include a cooperative network of multiple antioxidants that are compartmentalized to provide optimal protection against ROS-mediated oxidation. Previous studies showed that overexpression of a single antioxidant enzyme in skeletal muscle did not protect against contraction-induced oxidative damage to muscle fibres [35]. Therefore, regulation of upstream antioxidant signalling of Nrf2 with more generalised up-regulation of antioxidant defences may be a therapeutic approach against LPS induced preterm diaphragm weakness.
